# Impact of Sensor Data Characterization with Directional Nature of Fault and Statistical Feature Combination for Defect Detection on Roll-to-Roll Printed Electronics

**DOI:** 10.3390/s21248454

**Published:** 2021-12-18

**Authors:** Yoonjae Lee, Minho Jo, Gyoujin Cho, Changbeom Joo, Changwoo Lee

**Affiliations:** 1 Department of Mechanical Production Engineering and Design, Konkuk University, Seoul 05030, Korea; dldbswp913@konkuk.ac.kr (Y.L.); als8080@konkuk.ac.kr (M.J.); 2 Research Engineering Center for R2R Printed Flexible Computer, Department of Biophysics, Institute of Quantum Biophysics, Sungkyunkwan University, Suwon-si 16419, Korea; gcho1004@skku.edu; 3 Department of Mechanical Engineering, Stevens Institute of Technology, 1 Castle Point Terrace, Hoboken, NJ 07030, USA; cjoo@stevens.edu; 4 Department of Mechanical and Aerospace Engineering, Konkuk University, Seoul 05030, Korea

**Keywords:** defect detection, Directional Nature of Fault, gravure printing, fault diagnosis, roll-to-roll printed electronics, sensor data characterization

## Abstract

Gravure printing, which is a roll-to-roll printed electronics system suitable for high-speed patterning of functional layers have advantages of being applied to flexible webs in large areas. As each of the printing procedure from inking to doctoring followed by ink transferring and setting influences the quality of the pattern geometry, it is necessary to detect and diagnose factors causing the printing defects beforehand. Data acquisition with three triaxial acceleration sensors for fault diagnosis of four major defects such as doctor blade tilting fault was obtained. To improve the diagnosis performances, optimal sensor selection with Sensor Data Efficiency Evaluation, sensitivity evaluation for axis selection with Directional Nature of Fault and feature variable optimization with Feature Combination Matrix method was applied on the raw data to form a Smart Data. Each phase carried out on the raw data progressively enhanced the diagnosis results in contents of accuracy, positive predictive value, diagnosis processing time, and data capacity. In the case of doctor blade tilting fault, the diagnosis accuracy increased from 48% to 97% with decreasing processing time of 3640 s to 16 s and the data capacity of 100 Mb to 5 Mb depending on the input data between raw data and Smart Data.

## 1. Introduction

Roll-to-roll processing is highly advantageous because it results in multiple functional layers of electronic circuitry printed on large flexible materials (i.e., web) [1,2,3]. Gravure printing is the desirable mode for fabricating these printed electronic devices, owing to its characteristic high-speed patterning of component layers [4,5,6]. Gravure printing can be classified into the following four phases: inking, doctoring, ink transfer, and ink setting [7,8]. Printing defects can be generated by undesired printing conditions and ink characteristics during each printing phase [9,10,11,12]. For example, during the doctoring phase, the misalignment of the doctor blade at either side can degrade the ink uniformity in the engraved patterns in the width direction (i.e., transverse direction (TD)). Moreover, non-uniform nip roll pressure can negatively affect the uniformity of the pattern thickness in the TD. To derive high-quality patterns with uniform thickness using the roll-to-roll gravure printing process, it is necessary to recognize and diagnose these.

In this study, a method of data characterization using sensor data efficiency evaluation ($S_{E}$), directional nature of fault (DNF), and feature combination matrix (FCM) is proposed to diagnose these major faults. The aim is to recognize defects in advance and improve the diagnosis results by optimizing the training (input) data acquired from multiple sensors for the machine-learning fault diagnosis model. We find that the misalignment of the doctor blade, eccentricity of the nip and printing rolls, and non-uniform nip pressure can be indirectly measured via the vibration of the doctor blade, the nip roll, and the frames supporting the printing module. Through the acquisition of vibration data using multiple sensors, a vibration dataset (i.e., Raw data) is acquired. The smart data clearly show the characteristics of the vibration caused by the factors mentioned above, and they are selected from the raw dataset using the proposed methods in three phases to maximize performance efficiency. The evaluation criteria include diagnosis accuracy, positive predictive value (PPV), processing time for diagnosis, and data capacity. The performance of the machine-learning model developed using smart data was compared to that of the model just using the raw dataset.

With significant growth of industrial machines, recent studies have raised concerns regarding the maintenance of operating conditions. Profound interest in the fields of fault diagnosis based on data acquisition of sensors has been shown in recent research. Xia et al. presented convolutional neural network-based feature extraction approaches for fault diagnosis of rotating machines with multiple sensors [13]. Duan et al. have reviewed fields of fault diagnosis and condition monitoring based on multi-sensors for rolling bearings by presenting foundational knowledge [14]. Studies with multirate data and sensors for fault diagnosis by feature extracting deep learning models has been carried out by Zhao et al. and Huang et al. [15,16]. Research for fault diagnosis based on data optimization in recent studies has been shown by Bazan et al. and Wang et al. [17,18]. Lee et al. proposed quantification methods of fault features for rotary machine fault diagnosis. Most studies regarding fault diagnosis have shown methods of feature extraction to improve the results of machine learning from the data acquisition of sensors. 

As shown in studies abovementioned, diagnosing the abnormal conditions with multiple sensors show promising results of fault diagnosis; however, the efficiency of diagnosis performance is without consideration. As studies focus on methods or strategies to conclude in diagnosis, this paper proposes methods to optimize multiple sensor data by selecting an optimal sensor. Furthermore, in comparison with Bazan et al., the performances of diagnosis results regarding accuracy, and data reduction stretch to positive predictive value and diagnosis processing time [17]. Related to Lee et al., this paper proposes strategies based on quantification methods to evaluate the efficiency of each phase [19].

## 2. Methodology of Data Characterization

### 2.1. Procedure of Data Characerization from Raw Data to Smart Data

**Figure 1:**
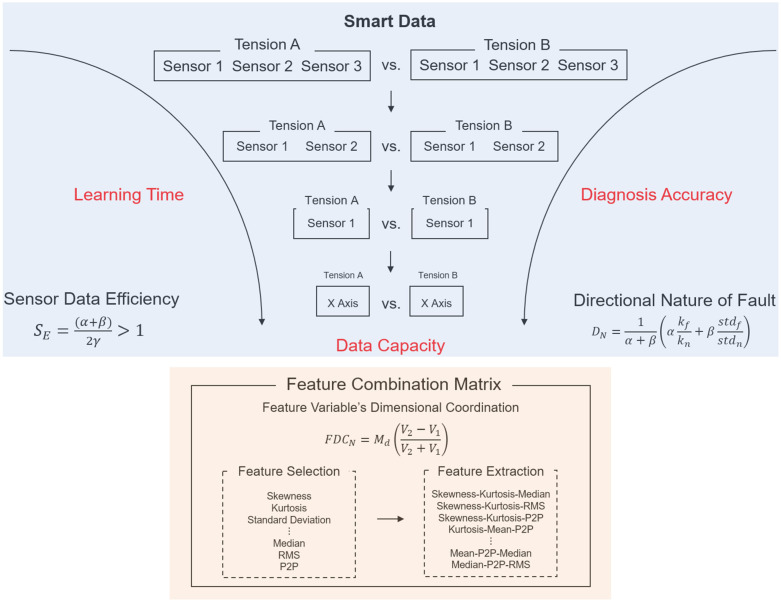
Smart data characterization procedure from raw data.

Procedure of data characterization is led with data acquisition with three acceleration sensors which are attached to the doctor’s blade and the frame of the gravure printing system. Each sensor is capable of acquiring data with three axes. Then on, experimentally acquired raw data is achieved in three phases, as shown in Figure 1. During Phase 1, the acquired sensor data are evaluated for efficiency ($S_{E}$), and the most efficient (optimal) sensor is chosen for DNF processing in Phase 2 to extract the most sensitive of three axes from the sensor. Then, a list of feature variables is tallied for training data using the FCM method in Phase 3. Finally, the processed smart data are used as input to the machine-learning fault diagnosis model to classify the printing process operating conditions during the major fault occurrences. Further description of smart data characterization through Phases 1–3 will be extensively illustrated in detail in Section 2.2, Section 2.3 and Section 2.4.

### 2.2. Sensor Data Efficiency Evaluation

The optimally efficient sensor is selected using an evaluation procedure based on Equation (1), which leverages three variables. α is the ratio of the data capacity between raw data and single-sensor data. β is the ratio of the data processing time, and γ is the ratio of the misclassification rate. Likewise, β and γ is a ratio between raw data and single-sensor data. Since the value of $S_{E}$ in Equation (1) is dependent on the ratio of three variables of two comparing data, the sensor rating the highest $S_{E}$ is selected as the optimal single sensor. In other words, a sensor with the clearest distinction to the raw data in three aspects abovementioned is likely to score the highest $S_{E}$.

(1) $$S_{E}=\frac{\alpha +\beta }{2\gamma }$$

In the case of this experiment, the diagnosis results from the raw data of three triaxial sensors were compared. 

### 2.3. Directional Nature of Fault

The DNF method extracts valid data from raw data by evaluating the sensitivity of the axial information from a single sensor. After Phase 1, the DNF method evaluates axes X, Y, and Z to extract valid data for fault diagnosis. The DNF method is defined in Equation (2), where α and β are weight factors defining the relative ratio between kurtosis and standard deviation. $k_{f}\;\mathrm{and}\;k_{n}$ are the kurtosis of the fault and normal conditions, respectively. $std_{f}\;\mathrm{and}\;std_{n}$ are the standard deviation of the fault and normal conditions, respectively. Based on the probability distribution curve, the standard deviation of the abnormal condition data has a wide distribution of data points [20,21]. The kurtosis of an abnormal condition has an imbalanced distribution [22]. The DNF number based on Equation (2) can thus be acquired from each axis. The axis with the highest DNF number defines the most sensitive and valid data for training.

(2) $$D_{N}=\frac{1}{\alpha +\beta }\left(\alpha \frac{k_{f}}{k_{n}}+\beta \frac{std_{f}}{std_{n}}\right)$$

### 2.4. Feature Combination Matrix

**Figure 2:**
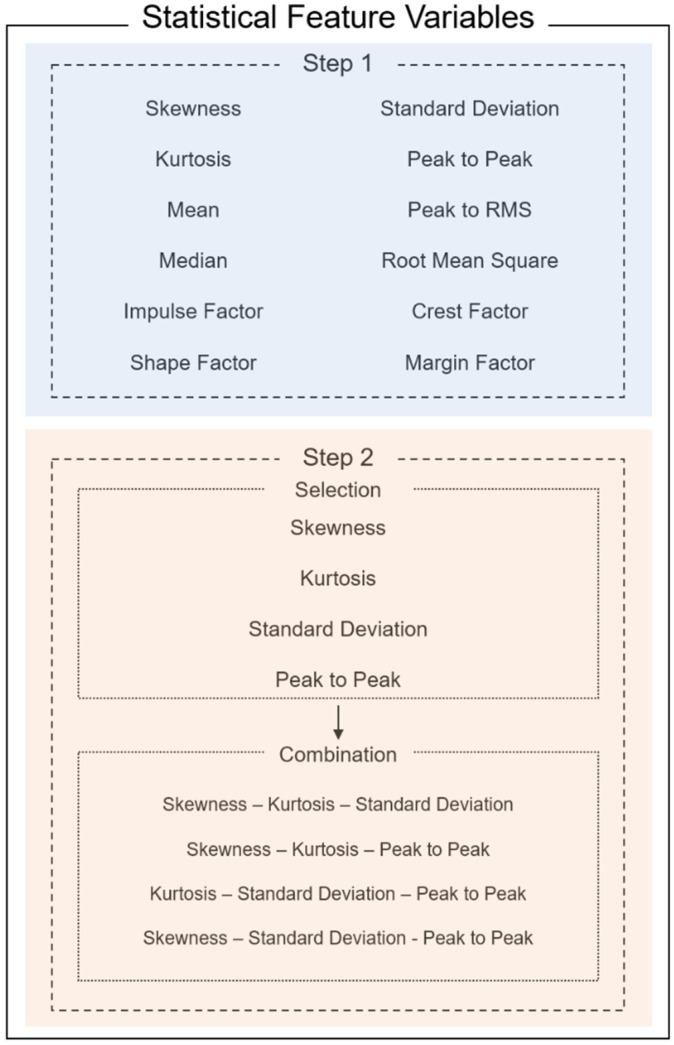
Feature engineering for feature combination matrix.

The FCM method selects and extracts statistical feature variables. As shown in Figure 2, feature extraction is performed when the list of statistical feature variables is acquired from the dataset from Phase 2 [23,24]. The extracted features are then combined into the three features of a three-dimensional volume. As mentioned in Section 2.3, based on a normal distribution, the distribution of data points is likely to be imbalanced, broad, skewed, or irregular [25,26,27]. Comparing the volume acquired from the combination of the three features, the volume of the normal condition data is smaller than that of the abnormal condition. Hence, the combination producing the largest difference between the two volumes of different conditions reflects higher classification accuracy. The distance between the two datasets is also a factor that improves classification performance because it distinguishes between normal and abnormal conditions. The Mahalanobis distance is applied to evaluate the distance between two datasets in a multivariate space, including correlated points for multiple variables, considering the densities of the datasets [28,29,30,31]. Using the volumes of normal/abnormal feature combinations and the Mahalanobis distance feature variables, the Feature Variable’s Dimensional Coordination number ($FDC_{N}$) can be obtained. As shown in Equation (3), the $FDC_{N}$ evaluates the combination of extracted features to ranks them according to efficiency. $V_{1}$ represents the volume of the normal condition feature combination, $V_{2}$ represents the volume of the abnormal condition feature combination, and $M_{d}$ represents the Mahalanobis distance between $V_{1}$ and $V_{2}$.

(3) $$FDC_{N}=M_{d}\left(\frac{V_{2}-V_{1}}{V_{2}+V_{1}}\right)\;$$

The selected feature combination through evaluation of the $FDC_{N}$ is then applied to be used as training data for developing a machine learning fault diagnosis model.

## 3. Experimental Data Acquisition

**Figure 3:**
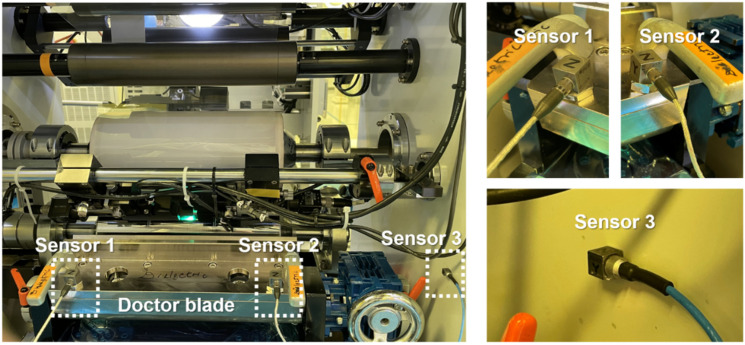
Experimental data acquisition by sensor position designation within the printing section.

The experimental data acquisition for major fault diagnosis of the gravure printing system is shown in Figure 3. As shown, acceleration Sensors 1, 2, and 3 were installed on both sides of the doctor blade and the frame supporting the printing module. All sensor outputs were obtained using a data acquisition module (NI-9230, National Instruments). Table 1 lists the specifications of the acceleration sensor and the NI-9230 module. When the sensors obtained the vibration data, they were transferred to the LabVIEW software to monitor and save the acquired data. 

**Figure 4:**
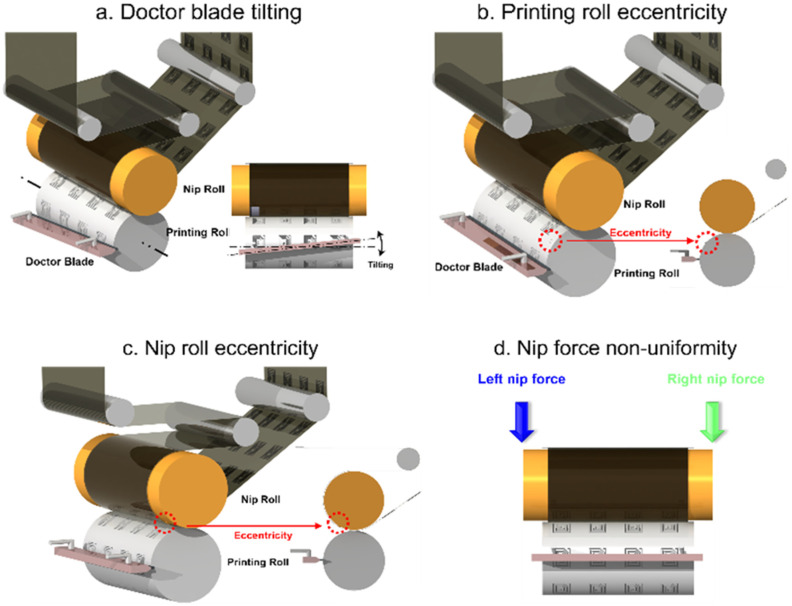
Possible main faults during gravure printing process: (**a**) Doctor blade tilting fault; (**b**) Printing roll eccentricity fault; (**c**) Nip roll eccentricity fault; and (**d**) Nip force non-uniformity fault.

The possible main faults during the printing process of the gravure printing system are shown in Figure 4. The four main faults of the experimental design include doctor blade tilting, printing roll eccentricity, nip roll eccentricity, and nip force non-uniformity. To detect the main faults for diagnosis, the experimental variables included the doctor blade, nip force, and tension. Cases with and without doctoring, and cases with and without nipping were tested under tensions of 2, 4, and 6 kgf. Regarding the nip force, the nipping cases were tested under 5 and 10 kgf, as shown in Table 2. 

**Figure 5:**
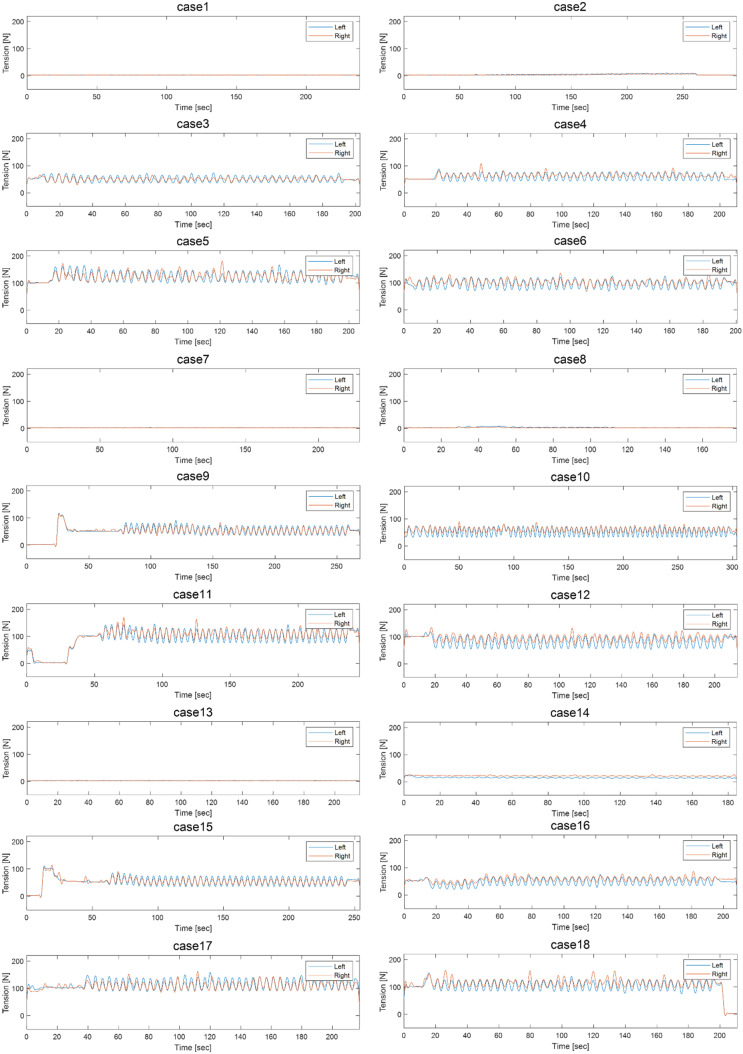
Nip force uniformity data of Cases 1–18.

As shown in Table 3, each case was tested under different tension, nip force, and doctoring conditions. The data used for diagnosing the doctor blade tilting fault required Cases 1 and 2 at an operating tension of 2 kgf, Cases 7 and 8 at an operating tension of 4 kgf, and Cases 13 and 14 at an operating tension of 6 kgf. Cases 1, 7, and 13 had different operating tensions; however, they were tested without and without doctoring. Cases 2, 8, and 14 also had different operating tensions with and without doctoring. The data for the fault diagnosis of the doctor blade tilting fault were acquired from the comparison of each case at the same operating tension. The data for diagnosis printing roll eccentricity were acquired from Cases 1, 7, and 13, which lack nipping and doctoring. Case comparison for nip roll eccentricity required conditions without doctoring; hence, Cases 5, 11, and 17 with a nip force of 10 kgf were compared to cases 1, 7, and 13. Nip force non-uniformity cases were selected using the nip force data shown in Figure 5. Cases 9 and 15 with uniform nip forces were compared to Cases 11 and 17. 

## 4. Results

### 4.1. Doctor Blade Tilting Fault Diagnosis

#### 4.1.1. Doctor Blade Tilting Fault Diagnosis Based on Raw Data

In this section, the fault diagnosis results of the doctor blade tilting fault based on the raw data are presented in Table 4. The raw data in this case include all data acquired from Sensors 1, 2, and 3. The diagnosis of a doctor blade tilting fault at an operating tension of 2 kgf showed 58.2 with a diagnosis accuracy of 1508.9 s and a processing capacity of 115 Mb. For a tension of 4 kgf, accuracy rates of 48.1% at 3640.4-s processing time required 100-Mb data capacity. At a tension of 6 kgf, the accuracy of fault diagnosis rates was 67.2%, which was the highest among tensions by 368.4 s with 113-Mb data size. 

#### 4.1.2. Optimal Sensor Selection Based on Sensor Efficiency Evaluation Method

The sensor data efficiency method described in Section 2.2 was applied to the raw data to select a single optimal sensor for performance improvement. Because the raw data comprised all sensor data, the sensor data efficiency method evaluates the sensors individually, as shown in Table S1. To evaluate the efficiency of $S_{E}$, the data capacity (α), processing time (β), and misclassification rate (γ) must be obtained from individual sensors. Sensors 1 and 2 from Figure 3 were evaluated because both were installed on the doctor blade in the same directions as the X, Y, and Z axes. Tables S1–S3 show the results of the sensor data efficiency evaluation, comparing the raw data to the data of Sensors 1 and 2. The results of the doctor blade tilting fault diagnosis for optimal sensor selection in Tables S1–S3 show that the highest $S_{E}$ results for Sensor 1 are as listed in Table 5. 

The result of the optimal sensor selection can be verified in Table S4 as compared with Table S5, based on the performance of the diagnosis results. It can also be seen that the diagnosis result of Sensor 1 was improved in accuracy, processing time, and data capacity compared with the result of raw data diagnosis shown in Table 4.

#### 4.1.3. Optimal Axis Selection Based on the DNF Method

Sensor 1 from the raw data of the doctor blade tilting cases was selected as the optimal sensor, and the DNF method was used to evaluate axes X, Y, and Z from Sensor 1 to extract the most sensitive axis. As mentioned in Section 2.3, based on the kurtosis and standard deviation of normal and abnormal conditions, the DNF number was calculated. The axis having the highest number of DNFs resulted in the highest diagnostic performance. As shown in Table 6, the DNF number evaluation of the X, Y, and Z axes from Sensor 1 is shown. As shown in Table 6, the axis having the highest DNF number differed depending on the operating tension. For a tension of 2 kgf, the Y-axis resulted in the highest DNF number. Tensions of 4 and 6 kgf showed the highest DNF numbers on the X-axis. The theory of achieving the highest diagnosis performance depending on the DNF number is verified in Tables S6–S8. Table S6 shows the highest accuracy of diagnosis for tensions of 4 and 6 kgf along the X-axis, and Table S7 illustrates the best result for a tension of 2 kgf. The proposed method evaluates the sensitivity of the axis using the DNF number, which resulted in a high rate of diagnosis accuracy and decreased processing time and data capacity requirements. 

#### 4.1.4. Feature Variable Optimization Based on FCM Method

**Figure 6:**
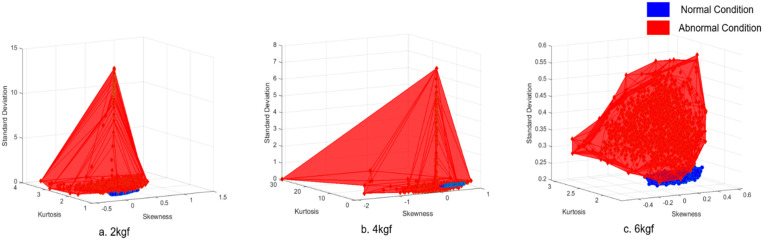
Volume comparison of normal and abnormal condition data: (**a**) Operating tension of 2 kgf; (**b**) Operating tension of 4 kgf; and (**c**) Operating tension of 6 kgf.

As shown in Figure 2, 12 feature variables were extracted from the data acquired during Phases 1 and 2. From the 12 feature variables, four were selected to be coordinated into a feature combination. The four variables in this case were skewness, kurtosis, standard deviation, and peak-to-peak. The left and right sides of the statistical feature variables are generally symmetrical around the mean on a normal distribution. Hence, skewness and kurtosis are selected as indicators to determine how far the distribution shape of the data deviates from normal. Skewness measures the asymmetry of the distribution. The more symmetric the data, the closer the skewness to zero. Furthermore, because kurtosis is a measure of outliers present in the distribution, there are clear criteria for discriminating between normal and abnormal, such as finding a value of three in the Gaussian probability distribution. In the case of peak-to-peak, peak vibration can be observed on the distribution chart when an abnormality occurs. Hence, the FCM method was applied to skewness, kurtosis, standard deviation, and peak-to-peak. The coordination of three feature variables of the selected four forms a volume, as shown in Figure 6. The red volume represents the three-dimensional feature variables of the abnormal condition data. The blue volume represents normal condition data. A significant volume difference between normal and abnormal conditions is visible. After evaluating the coordination of feature combinations from the selected feature variables using the FDC number from Equation (3), the combination having the highest FDC number was used as input data to train the machine-learning fault diagnosis model. As shown in Table 7, the fault diagnosis results of the doctor blade tilting condition improved, owing to the data characterization process of Phases 1, 2, and 3. Compared with the results of the raw data-based diagnosis in Table 4, the smart data-based fault diagnosis resulted in an improved accuracy of 90.1% from 58.2% at a tension of 2 kgf. At 4 kgf, the accuracy improved from 48.1% to 86.2%, and 67.2% to 97.0% at a tension of 6 kgf. The processing time reduced from 1508.9 s to 33.9 s at a tension of 2 kgf. It reduced from 3640.4 s to 37.5 s at 4 kgf. It reduced from 368.4 s to 16.6 s at 6-kgf tension. The data capacity was also reduced from approximately 113 Mb to 4 Mb. 

### 4.2. Printing Roll Eccentricity Fault Diagnosis

#### 4.2.1. Printing Roll Eccentricity Fault Diagnosis Based on Raw Data

The fault diagnosis of printing roll eccentricity was conducted using the raw data of processes at tensions of 2, 4, and 6 kgf, as listed in Table 3. As shown in Table 8, the results based on the raw data showed a diagnosis accuracy of 69.7–76.9%. The processing time of the raw data diagnosis ranged from 208.0 s to 237.9 s. 

#### 4.2.2. Printing Roll Eccentricity Fault Diagnosis Based on Smart Data

The diagnosis of the printing roll eccentricity fault data was performed in the same order as the doctor blade tilting diagnosis procedure described in Section 4.1. Based on the raw data of Phase 2, the sensor data efficiency evaluation was applied to select a single optimal sensor. As shown in Tables S9–S11, the data capacity, processing time, and misclassification rate of each case were computed to obtain $S_{E}$, as shown in Table 9. $S_{E}$ results of Sensor 2 reflected the highest value for all tensions. The fault diagnosis results based on Sensors 1 and 2 are shown in Tables S12 and S13 as applied to the verification of the sensor data efficiency evaluation. 

Based on the selected optimal Sensor 2 data, the DNF method was applied to extract the most sensitive axis information based on the DNF number. The results of the computation of the number of DNFs are listed in Table 10. The X-axis for tension 2 (4 kgf) resulted in the highest DNF number followed by the Z-axis for the remaining cases. Verification results of the selected axis depended on the cases based on the DNF number and are shown in Tables S14–S16. Compared with Table 10, the diagnostic performance of the selected axis having the highest DNF number provided the most efficient outcome. 

**Figure 7:**
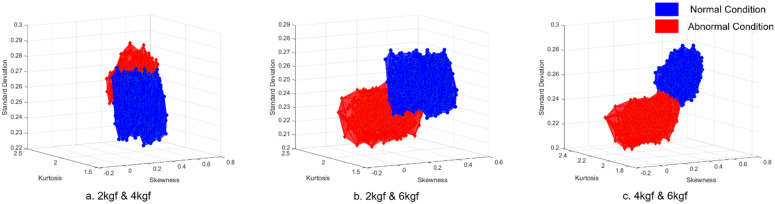
Volume comparison of normal and abnormal condition data: (**a**) Operating tensions of 2 and 4 kgf; (**b**) Operating tensions of 2 and 6 kgf; and (**c**) Operating tensions of 4 and 6 kgf.

As shown in Figure 7, the feature variables were extracted and combined into three feature combinations for evaluation. The selected and extracted feature variables were identical to those described in Section 4.1.4. The conditions of normal and abnormal data formed a volume measure for each feature variable, as shown in Figure 7. The two conditions were then computed using Equation (3) to select the training input data. Based on the results of the FCM method, it was then used as input data for printing roll eccentricity fault diagnosis. The results are listed in Table 11. Compared with Table 8, smart data increased the diagnosis accuracy up to 99.1% with a processing time of 3.7 s and a data capacity of 4 Mb. In summary, diagnosing the main fault printing roll eccentricity with smart data improved the diagnostic performance with less time consumption and fewer data requirements. 

### 4.3. Nip Roll Eccentricity Fault Diagnosis

#### 4.3.1. Nip Roll Eccentricity Fault Diagnosis Based on Raw Data

The fault diagnosis of the nip roll eccentricity based on raw data is shown in Table 12. The results for cases of tensions 2, 4, and 6 kgf rated 42.1% to 56.0% diagnosis accuracy with 425.4 s to 597.0 s of processing time. The data capacity of the raw data ranged from 111 Mb to 114 Mb, like the raw data capacity of doctor blade tilting and printing roll eccentricity faults. 

#### 4.3.2. Nip Roll Eccentricity Fault Diagnosis Based on Smart Data

The smart data transition from the raw data is presented in this section. The evaluation of the sensor data efficiency in Phase 1 used to select the optimal sensor is shown in Table 13. Sensor 1 was selected as the optimal sensor for the next phase of the DNF method. It can be seen that the $S_{E}$ of each case at Sensor 1 was higher than that of Sensor 2. As shown in Tables S17–S19, the data capacities of Sensors 1 and 2 maintained an average value of 43. As the capacity difference of both sensors merely influenced factor α, the major factor influencing the outcome of $S_{E}$ was at factors β and γ. Tables S20 and S21 verify that the sensor having the highest $S_{E}$ maintained the diagnosis result with higher accuracy. 

The evaluation of the X, Y, and Z axes of Sensor 1 was carried out based on the DNF method and the DNF number. The results from the most sensitive axis for each case are listed in Table 14. For the case of the tension of 2 kgf, the Z-axis rate had the highest $D_{N}$, whereas tensions of 4 and 6 kgf rates were the highest in the X-axis. The diagnosis results for each case, based on the axis of Sensor 1, are shown in Tables S22–S24. 

The FCM method was carried out based on the results of Phase 2 in this section. The feature variables used for coordination of the combination were identical to the results of Section 4.1 and Section 4.2 by skewness, kurtosis, standard deviation, and peak-to-peak. Kurtosis considers the effect of data at the end of the distribution on the probability curve. Based on the standard distribution, the kurtosis value increased with the weight of the outer values. Hence, kurtosis refers to the sharpness of the distribution, and if the degree of dispersion is large, the data are heterogeneous, and the height of the distribution is lowered. On the other hand, if the degree of dispersion is small, the data are homogeneous, and the height of the distribution increases. 

**Figure 8:**
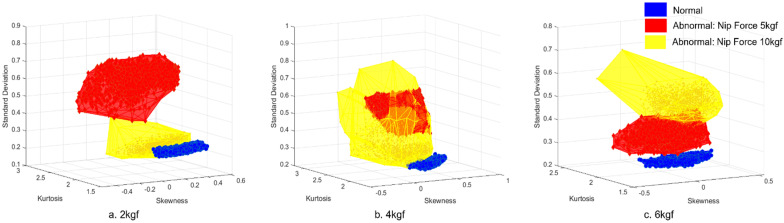
Volume comparison of normal and abnormal condition data: (**a**) Operating tension of 2 kgf; (**b**) Operating tension of 4 kgf; and (**c**) Operating tension of 6 kgf.

The volume of normal and abnormal conditions based on the coordinated feature variables can be seen in Figure 8. Normal volume is shown in blue, and abnormal volumes are shown in red and yellow. The abnormal volumes differ depending on the nip force of the data. Table 15 shows the results of the nip roll eccentricity fault diagnosis based on the smart data. In the case of the tension of 2 kgf, the diagnostic accuracy rates were 100% with a data capacity of 4 Mb and a processing time of 4.63 s. Compared with the results of the raw data in Table 12, it can be seen that the fault diagnosis model performances improved in areas of accuracy, positive predictive value, processing time, and data capacity. 

### 4.4. Nip Force Non-Uniformity Fault Diagnosis

#### 4.4.1. Nip Force Non-Uniformity Fault Diagnosis Based on Raw Data

Fault diagnosis based on raw data was performed to detect nip force non-uniformity. Figure 5 shows the data of the nip force for Cases 1–18. As Cases 11 and 17 in Figure 5 showed non-uniformity nip forces, the data of both cases were used as abnormal condition data for fault diagnosis. Table 16 shows the performance of the fault diagnosis at tensions of 4 and 6 kgf. 

#### 4.4.2. Nip Force Non-Uniformity Fault Diagnosis Based on Smart Data

The sensor data efficiency evaluation results are shown in Table 17 based on the computation of Tables S25 and S26. It can be seen that Sensor 2 had the highest $S_{E}$ among the raw data. Tables S27 and S28 can be used to verify the optimal sensor selection results of the sensor data efficiency evaluation. 

The DNF method was used to evaluate the axis of Sensor 2 by X, Y, and Z for tension cases of 4 and 6 kgf. The DNF numbers for both cases are shown in Table 18, where the result of a tension of 4 kgf showed axis Y as the most valid, and X for the tension case of 6 kgf. The results of the fault diagnosis based on Sensor 2 for the triaxis are shown in Tables S29–S31. 

**Figure 9:**
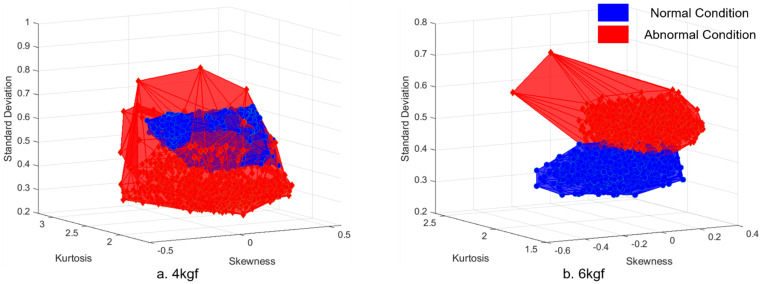
Volume comparison of normal and abnormal condition data: (**a**) Operating tension of 4 kgf; (**b**) Operating tension of 6 kgf.

With identical feature variables coordinated through the FCM method, the volumes of normal and abnormal conditions are shown in Figure 9. It can be seen from Figure 9a that the volume of the normal condition overlaps with the volume of the abnormal condition. Thus, the peak values and the distribution of data points for abnormal conditions were broad, compared with the normal volume condition. Based on the results of the FCM, the nip force non-uniformity fault diagnosis results with smart data are shown in Table 19. 

### 4.5. Simultaneous Fault Diagnosis

In Section 4.1, Section 4.2, Section 4.3 and Section 4.4, defects caused during the printing process of gravure printing system has been diagnosed independently. However, occasionally in real applications it is likely for the gravure printing system to malfunction with more than one single fault. In this section, characterized smart data has been applied under the assumption of multiple faults appearing simultaneously to present the effectiveness of the diagnosis model performance. 

Cases 6, 12, and 18 from Table 2 has been selected for the multiple fault data since the experimental condition included with nipping and doctoring at tensions 2, 4, and 6 kgf. Diagnosis results of simultaneous multiple faults is shown in Table 20. The effectiveness of the smart data characterization is shown with comparison to the diagnosis result with raw data. As the raw data of simultaneous faults contain various disturbances with noticeable peaks, it is less complex for the raw data-based diagnosis model to clarify the distinct conditions for classification. Hence, the average accuracy of raw data diagnosis is at 72.3% in which rates a higher value compared to single fault diagnosis results. Therefore, results based on smart data rates at an average of 99% on the grounds of abovementioned basis. In short, detecting simultaneous multiple faults based on smart data shows positive results as shown in Table 20. 

### 4.6. Raw Data and Smart Data Comparison for Fault Diagnosis

The fault diagnosis of four possible major faults during the printing process of the gravure printing system based on raw and smart data is shown in Table 21. Table 21 summarizes the impact of data characterization methods for the diagnosis of the four suggested major faults and the simultaneous faults of the gravure printing system printing process. The diagnosis performance comparison results are shown based on raw and smart data. All diagnosis results based on raw data and smart data are processed through support vector machine algorithm. In Tables S32–S35, diagnosis results of the four major faults depending on the machine learning algorithm is shown. A total of eight different algorithms have been applied to each of the faults and consequently shows that the most efficient outcome of the performance regarding accuracy, positive predictive value, and processing time concludes with the use of a support vector machine algorithm to diagnose all faults of the printing process. 

Based on the results of Table 21, techniques to increase the accuracy of the classification has been applied to faults of doctor blade tilting, printing roll eccentricity, and nip force non-uniformity. As the abovementioned faults maintain an accuracy of 97% to 99%, it is possible to improve the final diagnosis results by adjusting the parameter of window size. As shown is Equation (4), the window size can be adjusted using the sampling rate and revolutions per minute. As x is the revolutions per minute, and α as the sampling rate (Hz), it is possible to obtain the value $W_{s}$. Once the value $W_{s}$ is obtained for the three faults it is then applied to as a fixed parameter to be diagnosed based on the smart data. The results show in Table 22 that the contents of accuracy, PPV, and processing time have improved in comparison to the results of Table 21.

(4) $$W_{s}=\alpha {\left(\frac{x}{60}\right)}^{-1}\;$$

## 5. Conclusions

Printing defects generated by the misalignment of the doctor blade, eccentricity of the nip and printing rolls, and non-uniform nip roll pressures can negatively affect the performance of printed electronic devices. To prevent printing defects and to obtain high-quality printed functional layers, it is necessary to recognize and diagnose factors that cause printing defects. In this study, a method for data characterization using sensor data efficiency evaluation ($S_{E}$), DNF, and FCM methods was proposed to diagnose the possible four major faults in the roll-to-roll gravure printing process, followed by experimental verification. The misalignment of the doctor blade, printing roll eccentricity, nip roll eccentricity, nip force non-uniformity, and simultaneous faults rated an average value of 56% accuracy with raw data. However, with smart data, the accuracy rated 100.0% on average. The positive predictive value increased when the learning time was reduced from 1247 s to 12 s on average. The data capacity was reduced from 112 Mb to 5 Mb, depending on the selection of the sensor and its axis with optimized feature variable coordination. It is known that, with the use of smart data through sensor data efficiency evaluation, the feature combination matrix, and DNF methods, machine learning fault diagnosis model performance improves for classifying normal and abnormal conditions of datasets. The proposed smart data process in this paper is the most novel and contributory aspect of this paper because it leads to the near-perfect performance of the machine learning fault detection model. It is also faster and less computer-memory intensive than the results found from raw sensor data. This poses a contribution to the field, and countless industries can benefit from the improved and most cost-efficient production of printed electronics. Further research regarding the methodologies proposed in this paper plans to expand the application for fault diagnosis despite the numerous numbers of sensors.

## Figures and Tables

**Table 1:** Specifications of acceleration sensor and NI-9230 module.

Item	Parameter	Value
Sensor	Sensor Type	Share Accelerometer, Triaxial
	Sensitivity $[\mathrm{mV}/\left(m/s^{2}\right)$]	5.15
	Measurement Range $[m/s^{2}$]	±1000 g peak
	Frequency Range [Hz]	2–5000 Hz
	Resolution $[m/s^{2}$]	0.003 (rms)
Module	Sampling Rate Range [Hz]	0–1651.6
	Sampling Time per Trial [s]	36,000

**Table 2:** Experimental design of data acquisition with experimental variables of tension, nip force, and doctoring.

Case No.	Tension [kgf]	Nip Force [kgf]	Doctoring
1	2	Without Nipping	Without Doctoring
2		Without Nipping	With Doctoring
3		5	Without Doctoring
4		5	With Doctoring
5		10	Without Doctoring
6		10	With Doctoring
7	4	Without Nipping	Without Doctoring
8		Without Nipping	With Doctoring
9		5	Without Doctoring
10		5	With Doctoring
11		10	Without Doctoring
12		10	With Doctoring
13	6	Without Nipping	Without Doctoring
14		Without Nipping	With Doctoring
15		5	Without Doctoring
16		5	With Doctoring
17		10	Without Doctoring
18		10	With Doctoring

**Table 3:** Case comparison for fault diagnosis of possible main faults during printing process of gravure printing system.

Case No.	Tension [2 kgf]	Tension [4 kgf]	Tension [6 kgf]
Doctor Blade Tilting	Case 1 vs. Case 2	Case 7 vs. Case 8	Case 13 vs. Case 14
Printing Roll Eccentricity	Case 1	Case 7	Case 13
Nip Roll Eccentricity	Case 1 vs. Case 5	Case 7 vs. Case 11	Case 13 vs. Case 17
Nip Force Non-Uniformity	-	Case 9 vs. Case 11	Case 15 vs. Case 17

**Table 4:** Doctor blade tilting fault diagnosis based on raw data (i.e., Sensors 1, 2, and 3).

[SVM]	2 kgf	4 kgf	6 kgf
Accuracy [%]	58.2	48.1	67.2
Positive Predictive Value [%]	51.4	36.7	64.4
Processing Time [s]	1508.9	3640.4	368.4
Data Capacity [Mb]	115	100	113

**Table 5:** Result of sensor data efficiency evaluation for optimal sensor selection of doctor blade tilting fault.

$S_{E}$	Sensor 1	Sensor 2
2 kgf	6	5.79
4 kgf	6.69	5.70
6 kgf	8.17	5

**Table 6:** DNF Number of axis X, Y, and Z from Sensor 1 of doctor blade tilting fault.

$D_{N}$	X Axis	Y Axis	Z Axis
2 kgf	8.27	11.66	6.00
4 kgf	16.76	15.53	8.64
6 kgf	1.59	1.32	1.01

**Table 7:** Doctor blade tilting fault diagnosis based on smart data.

[SVM]	2 kgf [Y Axis]	4 kgf [X Axis]	6 kgf [X Axis]
Accuracy [%]	90.1	86.2	97.0
Positive Predictive Value [%]	89.8	85.9	97.0
Processing Time [s]	33.9	37.5	16.6
Data Capacity [Mb]	5	4	5

**Table 8:** Printing roll eccentricity diagnosis based on raw data (i.e., Sensors 1, 2, and 3).

[SVM]	2 and 4 kgf	2 and 6 kgf	4 and 6 kgf
Accuracy [%]	74.8	76.9	69.7
Positive Predictive Value [%]	70.2	73.5	54.2
Processing Time [s]	237.9	208.0	237.0
Data Capacity [Mb]	111	111	110

**Table 9:** Result of sensor data efficiency evaluation for optimal sensor selection of printing roll eccentricity fault.

$S_{E}$	Sensor 1	Sensor 2
2 and 4 kgf	24.07	29.29
2 and 6 kgf	20.29	25.15
4 and 6 kgf	19.09	27.24

**Table 10:** DNF Number of axis X, Y, and Z from Sensor 2 of printing roll eccentricity fault.

$D_{N}$	X-Axis	Y-Axis	Z-Axis
2 kgf	1.12	0.91	1.06
4 kgf	1.09	0.86	1.12
6 kgf	0.95	1.01	1.06

**Table 11:** Printing roll eccentricity fault diagnosis based on smart data.

[SVM]	2 and 4 kgf [X Axis]	2 and 6 kgf [Z Axis]	4 and 6 kgf [Z Axis]
Accuracy [%]	97.9	99.1	96.3
Positive Predictive Value [%]	93.4	94.9	92.0
Processing Time [s]	6.1	5.1	3.7
Data Capacity [Mb]	5	5	4

**Table 12:** Nip roll eccentricity fault diagnosis based on raw data (i.e., Sensors 1, 2, and 3).

[SVM]	2 kgf	4 kgf	6 kgf
Accuracy [%]	53.8	56.0	42.1
Positive Predictive Value [%]	46.7	47.7	33.9
Processing Time [s]	425.4	574.4	597.0
Data Capacity [Mb]	111	111	114

**Table 13:** Result of sensor data efficiency evaluation for optimal sensor selection of nip roll eccentricity fault.

$S_{E}$	Sensor 1	Sensor 2
2 kgf	24.16	19.6
4 kgf	14.54	13.82
6 kgf	19.5	16.88

**Table 14:** DNF Number of axis X, Y, and Z from Sensor 1 of nip roll eccentricity fault.

$D_{N}$	X Axis	Y Axis	Z Axis
2 kgf	0.89	0.86	1.09
4 kgf	1.53	1.30	1.04
6 kgf	1.67	1.15	0.92

**Table 15:** Nip roll eccentricity fault diagnosis based on smart data.

[SVM]	2 kgf [Z Axis]	4 kgf [X Axis]	6 kgf [X Axis]
Accuracy [%]	100.0	98.4	99.5
Positive Predictive Value [%]	98.8	97.0	98.2
Processing Time [s]	4.63	4.38	4.40
Data Capacity [Mb]	4	4	4

**Table 16:** Nip force non-uniformity fault diagnosis based on raw data (i.e., Sensors 1, 2, and 3).

[SVM]	4 kgf	6 kgf
Accuracy [%]	65.5	65.4
Positive Predictive Value [%]	60.3	59.4
Processing Time [s]	281.7	515.4
Data Capacity [Mb]	115	116

**Table 17:** Result of sensor data efficiency evaluation for optimal sensor selection of nip force non-uniformity fault.

$S_{E}$	Sensor 1	Sensor 2
4 kgf	11.29	11.83
6 kgf	11.91	12.45

**Table 18:** DNF Number of X, Y, and Z axes from Sensor 2 of nip force non-uniformity fault.

$D_{N}$	X Axis	Y Axis	Z Axis
4 kgf	0.97	1.12	1.10
6 kgf	1.16	1.03	1.04

**Table 19:** Nip force non-uniformity fault diagnosis based on smart data.

[SVM]	4 kgf [Y Axis]	6 kgf [X Axis]
Accuracy [%]	97.9	95.2
Positive Predictive Value [%]	93.5	90.7
Processing Time [s]	25.4	28.4
Data Capacity [Mb]	6	5

**Table 20:** Simultaneous fault diagnosis result based on big data and smart data.

[SVM].	2 kgf [X Axis]	4 kgf [Y Axis]	6 kgf [Y Axis]
Accuracy [%]	70→97	74→100	73→100
Positive Predictive Value [%]	69→95	72→99	73→99
Processing Time [s]	4501→52	4035→34	4722→49
Data Capacity [Mb]	110→6	112→8	114→7

**Table 21:** Raw data and smart data diagnosis comparison.

Main Faults	Accuracy [%]	PPV [%]	Processing Time [s]	Data Capacity [Mb]
Doctor Blade Tilting	48→97	36→97	3640→16	110→5
Printing Roll Eccentricity	69→99	54→94	237→5	110→5
Nip Roll Eccentricity	42→100	33→98	597→4	114→4
Nip Force Non-Uniformity	65→97	59→93	515→25	116→6
Simultaneous Faults	74→100	72→99	4035→34	112→8

**Table 22:** Smart data diagnosis improvement with window size adjustment.

Main Faults	Accuracy [%]	PPV [%]	Processing Time [s]	Data Capacity [Mb]
Doctor Blade Tilting	48→100	36→99	3640→13	110→5
Printing Roll Eccentricity	69→100	54→99	237→8	110→5
Nip Force Non-Uniformity	65→100	59→98	515→17	116→6

## Data Availability

Not applicable.

## Supplementary Materials

The following are available online at https://www.mdpi.com/article/10.3390/s21248454/s1, Table S1: Sensor data efficiency evaluation of doctor blade tilting fault under operating tension 2 kgf, Table S2. Sensor data efficiency evaluation of doctor blade tilting fault under operating tension 4 kgf, Table S3. Sensor data efficiency evaluation of doctor blade tilting fault under operating tension 6 kgf, Table S4. Doctor blade tilting fault diagnosis result based on sensor 1, Table S5. Doctor blade tilting fault diagnosis result based on sensor 2, Table S6. Doctor blade tilting fault diagnosis result based on X axis of sensor 1, Table S7. Doctor blade tilting fault diagnosis result based on Y axis of sensor 1, Table S8. Doctor blade tilting fault diagnosis result based on Z axis of sensor 1, Table S9. Sensor data efficiency evaluation of printing roll eccentricity fault under case 2 kgf and 4 kgf, Table S10. Sensor data efficiency evaluation of printing roll eccentricity fault under case 2 kgf and 6 kgf, Table S11. Sensor data efficiency evaluation of printing roll eccentricity fault under case 4 kgf and 6 kgf, Table S12. Printing roll eccentricity fault diagnosis result based on sensor 1, Table S13. Printing roll eccentricity fault diagnosis result based on sensor 2, Table S14. Printing roll eccentricity fault diagnosis result based on X axis of sensor 2, Table S15. Printing roll eccentricity fault diagnosis result based on Y axis of sensor 2, Table S16. Printing roll eccentricity fault diagnosis result based on Z axis of sensor 2, Table S17. Sensor data efficiency evaluation of nip roll eccentricity fault under case 2 kgf, Table S18. Sensor data efficiency evaluation of nip roll eccentricity fault under case 4 kgf, Table S19. Sensor data efficiency evaluation of nip roll eccentricity fault under case 6 kgf, Table S20. Nip roll eccentricity fault diagnosis result based on sensor 1, Table S21. Nip roll eccentricity fault diagnosis result based on sensor 2, Table S22. Nip roll eccentricity fault diagnosis result based on X axis of sensor 1, Table S23. Nip roll eccentricity fault diagnosis result based on Y axis of sensor 1, Table S24. Nip roll eccentricity fault diagnosis result based on Z axis of sensor 1, Table S25. Sensor data efficiency evaluation of nip force non-uniformity fault under case 4 kgf, Table S26. Sensor data efficiency evaluation of nip force non-uniformity fault under case 6 kgf, Table S27. Nip force non-uniformity fault diagnosis result based on sensor 1, Table S28. Nip force non-uniformity fault diagnosis result based on sensor 2, Table S29. Nip force non-uniformity fault diagnosis result based on X axis of sensor 2, Table S30. Nip force non-uniformity fault diagnosis result based on Y axis of sensor 2, Table S31. Nip force non-uniformity fault diagnosis result based on Z axis of sensor 2, Table S32. Doctor blade tilting fault diagnosis with various machine learning algorithms, Table S33. Printing roll eccentricity fault diagnosis with various machine learning algorithms, Table S34. Nip roll eccentricity fault diagnosis with various machine learning algorithms, Table S35. Nip force non-uniformity fault diagnosis with various machine learning algorithms.
