# Supplementary material for: Impact of Sensor Data Characterization with Directional Nature of Fault and Statistical Feature Combination for Defect Detection on Roll-to-Roll Printed Electronics
Source: Sensors (Basel). 2021 Dec 18;21(24):8454. doi: 10.3390/s21248454 (PMC8706900; doi:10.3390/s21248454)
Supplement: Supplementary file 1 [file sensors-21-08454-s001.zip › sensors-1494786-supplementary.pdf]

sensors MDPI

Supplementary Materials

Table S1. Sensor data efficiency evaluation of doctor blade tilting fault under operating tension 2kgf.

[SVM]	Raw data	Sensor 1	Sensor 2	Value
Data Capacity [Mb] (α)	115	44	47	$\alpha_{S1} = 2.61$ $\alpha_{S2} = 2.44$
Processing Time [sec] (β)	1508.9	518.0	521.5	$\beta_{S1} = 2.91$ $\beta_{S2} = 2.89$
Misclassification Rate [%] (γ)	41.8	19.3	19.4	$\gamma_{S1} = 0.46$ $\gamma_{S2} = 0.46$

Table S2. Sensor data efficiency evaluation of doctor blade tilting fault under operating tension 4kgf.

[SVM]	Raw data	Sensor 1	Sensor 2	Value
Data Capacity [Mb] (α)	100	40	42	$\alpha_{S1} = 2.50$ $\alpha_{S2} = 2.38$
Processing Time [sec] (β)	6340.4	1339.4	1507.6	$\beta_{S1} = 2.72$ $\beta_{S2} = 2.41$
Misclassification Rate [%] (γ)	51.9	20.2	21.7	$\gamma_{S1} = 0.39$ $\gamma_{S2} = 0.42$

Table S3. Sensor data efficiency evaluation of doctor blade tilting fault under operating tension 6kgf.

[SVM]	Raw data	Sensor 1	Sensor 2	Value
Data Capacity [Mb] (α)	113	44	48	$\alpha_{S1} = 2.56$ $\alpha_{S2} = 2.35$
Processing Time [sec] (β)	368.4	85.7	88.7	$\beta_{S1} = 4.30$ $\beta_{S2} = 4.15$
Misclassification Rate [%] (γ)	32.8	13.9	21.3	$\gamma_{S1} = 0.42$ $\gamma_{S2} = 0.65$

Table S4. Doctor blade tilting fault diagnosis result based on sensor 1

**[SVM]**	**2kgf**	**4kgf**	**6kgf**
Accuracy [%]	**80.7**	**79.8**	**86.1**
Processing Time [sec]	**518.0**	**1339.4**	**85.7**
Data Capacity [Mb]	**44**	**40**	**44**

Table S5. Doctor blade tilting fault diagnosis result based on sensor 2

**[SVM]**	2kgf	4kgf	6kgf
Accuracy [%]	80.6	78.3	86.0
Processing Time [sec]	521.5	1507.6	88.7
Data Capacity [Mb]	47	42	48

Table S6. Doctor blade tilting fault diagnosis result based on X axis of sensor 1

**[SVM]**	2kgf	**4kgf**	**6kgf**
Accuracy [%]	85.7	**84.0**	**91.3**
Processing Time [sec]	264.6	**495.7**	**31.6**
Data Capacity [Mb]	15	**13**	**15**

Table S7. Doctor blade tilting fault diagnosis result based on Y axis of sensor 1

**[SVM]**	**2kgf**	4kgf	6kgf
Accuracy [%]	**86.9**	83.6	91.1
Processing Time [sec]	**254.6**	512.8	35.0
Data Capacity [Mb]	**14**	14	14

Table S8. Doctor blade tilting fault diagnosis result based on Z axis of sensor 1

**[SVM]**	2kgf	4kgf	6kgf
Accuracy [%]	82.0	81.3	89.8
Processing Time [sec]	289.3	527.7	42.4
Data Capacity [Mb]	15	13	15

Table S9. Sensor data efficiency evaluation of printing roll eccentricity fault under case 2kgf and 4kgf.

[SVM]	Raw data	Sensor 1	Sensor 2	Value
Data Capacity [Mb] (α)	111	39	41	$\alpha_{S1} = 2.85$<br>$\alpha_{S2} = 2.71$
Processing Time [sec] (β)	237.9	15.9	15.4	$\beta_{S1} = 14.96$<br>$\beta_{S2} = 15.45$
Misclassification Rate [%] (γ)	25.2	9.3	7.7	$\gamma_{S1} = 0.37$<br>$\gamma_{S2} = 0.31$

Table S10. Sensor data efficiency evaluation of printing roll eccentricity fault under case 2kgf and 6kgf.

[SVM]	Raw data	Sensor 1	Sensor 2	Value
Data Capacity [Mb] (α)	111	42	46	$\alpha_{S1} = 2.64$<br>$\alpha_{S2} = 2.41$
Processing Time [sec] (β)	208.0	18.0	16.4	$\beta_{S1} = 11.56$<br>$\beta_{S2} = 12.68$
Misclassification Rate [%] (γ)	23.1	8.2	6.9	$\gamma_{S1} = 0.35$<br>$\gamma_{S2} = 0.30$

Table S11. Sensor data efficiency evaluation of printing roll eccentricity fault under case 4kgf and 6kgf.

[SVM]	Raw data	Sensor 1	Sensor 2	Value
Data Capacity [Mb] (α)	110	37	41	$\alpha_{S1} = 2.97$<br>$\alpha_{S2} = 2.68$
Processing Time [sec] (β)	237.0	14.7	12.4	$\beta_{S1} = 16.12$<br>$\beta_{S2} = 19.11$
Misclassification Rate [%] (γ)	30.3	15	12.2	$\gamma_{S1} = 0.50$<br>$\gamma_{S2} = 0.40$

Table S12. Printing roll eccentricity fault diagnosis result based on sensor 1

**[SVM]**	2kgf and 4kgf	2kgf and 6kgf	4kgf and 6kgf
Accuracy [%]	90.7	91.8	85.0
Processing Time [sec]	15.9	18.0	14.7
Data Capacity [Mb]	39	42	37

Table S13. Printing roll eccentricity fault diagnosis result based on sensor 2

**[SVM]**	**2kgf and 4kgf**	**2kgf and 6kgf**	**4kgf and 6kgf**
Accuracy [%]	**82.3**	**93.1**	**87.8**
Processing Time [sec]	**15.4**	**16.4**	**12.4**
Data Capacity [Mb]	**41**	**46**	**41**

Table S14. Printing roll eccentricity fault diagnosis result based on X axis of sensor 2

**[SVM]**	**2kgf and 4kgf**	2kgf and 6kgf	4kgf and 6kgf
Accuracy [%]	**94.8**	95.4	90.6
Processing Time [sec]	**8.3**	10.5	10.3
Data Capacity [Mb]	**13**	15	14

Table S15. Printing roll eccentricity fault diagnosis result based on Y axis of sensor 2

**[SVM]**	2kgf and 4kgf	2kgf and 6kgf	4kgf and 6kgf
Accuracy [%]	93.4	94.2	92.4
Processing Time [sec]	10.1	12.9	7.0
Data Capacity [Mb]	13	16	14

Table S16. Printing roll eccentricity fault diagnosis result based on Z axis of sensor 2

**[SVM]**	2kgf and 4kgf	**2kgf and 6kgf**	**4kgf and 6kgf**
Accuracy [%]	94.0	**96.0**	**92.7**
Processing Time [sec]	9.9	**9.7**	**5.7**
Data Capacity [Mb]	15	**15**	**13**

Table S17. Sensor data efficiency evaluation of nip roll eccentricity fault under case 2kgf

[SVM]	Raw data	Sensor 1	Sensor 2	Value
Data Capacity [Mb] (α)	111	42	45	$\alpha_{S1} = 2.64$<br>$\alpha_{S2} = 2.47$
Processing Time [sec] (β)	425.4	117.1	124.7	$\beta_{S1} = 3.63$<br>$\beta_{S2} = 3.41$
Misclassification Rate [%] (γ)	46.2	6.2	7.0	$\gamma_{S1} = 0.13$<br>$\gamma_{S2} = 0.15$

Table S18. Sensor data efficiency evaluation of nip roll eccentricity fault under case 4kgf

[SVM]	Raw data	Sensor 1	Sensor 2	Value
Data Capacity [Mb] (α)	111	39	42	$\alpha_{S1} = 2.85$<br>$\alpha_{S2} = 2.64$
Processing Time [sec] (β)	574.4	130.1	134.4	$\beta_{S1} = 4.42$<br>$\beta_{S2} = 4.27$
Misclassification Rate [%] (γ)	44	10.8	11.1	$\gamma_{S1} = 0.25$<br>$\gamma_{S2} = 0.25$

Table S19. Sensor data efficiency evaluation of nip roll eccentricity fault under case 6kgf

[SVM]	Raw data	Sensor 1	Sensor 2	Value
Data Capacity [Mb] (α)	114	43	48	$\alpha_{S1} = 2.65$<br>$\alpha_{S2} = 2.38$
Processing Time [sec] (β)	597.0	186.6	198.0	$\beta_{S1} = 3.20$<br>$\beta_{S2} = 3.02$
Misclassification Rate [%] (γ)	57.9	8.6	9.1	$\gamma_{S1} = 0.15$<br>$\gamma_{S2} = 0.16$

Table S20. Nip roll eccentricity fault diagnosis result based on sensor 1

**[SVM]**	**2kgf**	**4kgf**	**6kgf**
Accuracy [%]	**93.8**	**89.2**	**91.4**
Processing Time [sec]	**117.1**	**130.1**	**186.6**
Data Capacity [Mb]	**42**	**39**	**43**

Table S21. Nip roll eccentricity fault diagnosis result based on sensor 2

**[SVM]**	2kgf	4kgf	6kgf
Accuracy [%]	93.0	88.9	90.9
Processing Time [sec]	124.7	134.4	198.0
Data Capacity [Mb]	45	42	48

Table S22. Nip roll eccentricity fault diagnosis result based on X axis of sensor 1

**[SVM]**	2kgf	**4kgf**	**6kgf**
Accuracy [%]	95.2	**92.4**	**94.5**
Processing Time [sec]	40.0	**45.7**	**58.8**
Data Capacity [Mb]	14	**13**	**14**

Table S23. Nip roll eccentricity fault diagnosis result based on Y axis of sensor 1

**[SVM]**	2kgf	4kgf	6kgf
Accuracy [%]	94.1	92.2	94.5
Processing Time [sec]	44.4	44.7	68.6
Data Capacity [Mb]	14	12	15

Table S24. Nip roll eccentricity fault diagnosis result based on Z axis of sensor 1

**[SVM]**	**2kgf**	4kgf	6kgf
Accuracy [%]	**96.8**	92.2	92.9
Processing Time [sec]	**35.7**	44.9	75.0
Data Capacity [Mb]	**14**	14	14

Table S25. Sensor data efficiency evaluation of nip force non-uniformity fault under case 4kgf

[SVM]	Raw data	Sensor 1	Sensor 2	Value
Data Capacity [Mb] (α)	115	45	48	$\alpha_{S1} = 2.56$ $\alpha_{S2} = 2.40$
Processing Time [sec] (β)	281.7	55.0	52.1	$\beta_{S1} = 5.12$ $\beta_{S2} = 5.41$
Misclassification Rate [%] (γ)	34.5	11.8	11.3	$\gamma_{S1} = 0.34$ $\gamma_{S2} = 0.33$

Table S26. Sensor data efficiency evaluation of nip force non-uniformity fault under case 6kgf

[SVM]	Raw data	Sensor 1	Sensor 2	Value
Data Capacity [Mb] (α)	116	44	48	$\alpha_{S1} = 2.64$ $\alpha_{S2} = 2.42$
Processing Time [sec] (β)	515.4	72.3	70.7	$\beta_{S1} = 7.13$ $\beta_{S2} = 7.29$
Misclassification Rate [%] (γ)	34.6	14.2	13.4	$\gamma_{S1} = 0.41$ $\gamma_{S2} = 0.39$

Table S27. Nip force non-uniformity fault diagnosis result based on sensor 1

**[SVM]**	4kgf	6kgf
Accuracy [%]	88.2	85.8
Processing Time [sec]	55.0	72.3
Data Capacity [Mb]	45	44

Table S28. Nip force non-uniformity fault diagnosis result based on sensor 2

**[SVM]**	**4kgf**	**6kgf**
Accuracy [%]	**88.7**	**86.6**
Processing Time [sec]	**52.1**	**70.7**
Data Capacity [Mb]	**48**	**48**

Table S29. Nip force non-uniformity fault diagnosis result based on X axis of sensor 2

**[SVM]**	4kgf	**6kgf**
Accuracy [%]	90.3	**90.7**
Processing Time [sec]	31.6	**44.4**
Data Capacity [Mb]	16	**14**

Table S30. Nip force non-uniformity fault diagnosis result based on Y axis of sensor 2

**[SVM]**	**4kgf**	6kgf
Accuracy [%]	**91.4**	90.0
Processing Time [sec]	**27.0**	47.5
Data Capacity [Mb]	**16**	15

Table S31. Nip force non-uniformity fault diagnosis result based on Z axis of sensor 2

**[SVM]**	4kgf	6kgf
Accuracy [%]	91.0	90.2
Processing Time [sec]	24.6	46.2
Data Capacity [Mb]	16	15

Table S32. Doctor blade tilting fault diagnosis with various machine learning algorithms

Algorithm	Accuracy [%]	Positive Predictive Value [%]	Processing Time [sec]
Bayesian Logistic Regression	93.1	93.0	27.0
Boosted Logistic Regression	90.4	90.2	32.1
K-Nearest Neighbor	90.6	90.1	30.7
Linear Discriminant Analysis	95.8	94.5	19.5
Logistic Regression	87.6	96.6	40.8
Naïve Bayes	88.7	88.5	40.1
Random Forest	84.5	82.7	39.8
**Support Vector Machine**	**97.0**	**97.0**	**16.6**

Table S33. Printing roll eccentricity fault diagnosis with various machine learning algorithms

Algorithm	Accuracy [%]	Positive Predictive Value [%]	Processing Time [sec]
Bayesian Logistic Regression	88.7	88.5	14.0
Boosted Logistic Regression	84.5	84.5	17.8
K-Nearest Neighbor	94.1	93.7	8.0
Linear Discriminant Analysis	97.9	96.7	7.8
Logistic Regression	72.6	72.4	40.0
Naïve Bayes	83.3	83.0	19.4
Random Forest	90.7	90.0	5.7
**Support Vector Machine**	**99.1**	**94.9**	**5.1**

Table S34. Nip roll eccentricity fault diagnosis with various machine learning algorithms

Algorithm	Accuracy [%]	Positive Predictive Value [%]	Processing Time [sec]
Bayesian Logistic Regression	94.4	94.0	4.7
Boosted Logistic Regression	97.0	95.5	5.7
K-Nearest Neighbor	91.4	90.8	11.0
Linear Discriminant Analysis	74.1	73.1	37.4
Logistic Regression	90.3	90.3	13.3
Naïve Bayes	89.0	88.4	27.1
Random Forest	99.2	94.5	22.9
**Support Vector Machine**	**100.0**	**98.8**	**4.6**

Table S35. Nip force non-uniformity fault diagnosis with various machine learning algorithms

Algorithm	Accuracy [%]	Positive Predictive Value [%]	Processing Time [sec]
Bayesian Logistic Regression	67.5	67.0	78.1
Boosted Logistic Regression	61.3	58.1	60.8
K-Nearest Neighbor	95.8	94.5	28.4
Linear Discriminant Analysis	92.0	92.0	20.7
Logistic Regression	70.7	66.8	72.0
Naïve Bayes	94.9	93.4	25.1
Random Forest	98.3	97.7	40.8
**Support Vector Machine**	**97.9**	**93.5**	**25.4**
